# Illness course variables as predictors of efficacy and speed of response to Spravaro in major depressive disorder

**DOI:** 10.1192/j.eurpsy.2026.11415

**Published:** 2026-07-31

**Authors:** R. G. Labrador, M. D. I. Ibañez de Gauna, M. R. A. Diaz

**Affiliations:** 1 Psiquiatria, Hupital Universitario Infanta Sofia; 2 Psiquiatria, Fundacion para la investigacion bimedica hospital 12 de Octubre, madrid, Spain

## Abstract

**Introduction:**

The response to antidepressant treatment may be influenced by clinical factors related to the course of major depressive disorder (MDD). Spravaro has emerged as an innovative therapeutic option; however, it remains unclear which variables determine its efficacy and speed of action.

**Objectives:**

To assess whether illness duration, number of previous depressive episodes, duration of depressive episodes, and duration of the last depressive episode are associated with treatment response and speed of response to Spravaro.

**Methods:**

We conducted an observational study including 44 patients diagnosed with MDD (30 women, 14 men; mean age 53.68 years). Spravaro was administered in a community mental health center, which we consider the most appropriate environment due to patient familiarity and the continuity of care provided by their own clinical team. Clinical response was assessed using the Clinical Global Impression (CGI) scale and a Likert scale evaluating subjective perception of improvement. Assessments were performed at 1 month, 3 months, 6 months, and at the end of treatment. Statistical analyses were conducted using SPSS Statistics v25.

**Results:**

Preliminary analyses suggest that illness duration and the length of the last depressive episode are associated with faster response according to CGI. Subjective perception of improvement showed greater interindividual variability. The number of depressive episodes appeared to influence the magnitude of clinical response.

**Conclusions:**

Clinical variables reflecting the natural history of depression—particularly illness duration and episode length—may modulate treatment outcomes with Spravaro. Administering treatment in community mental health centers, within the patient’s familiar environment and with their own care team, may further enhance adherence and treatment optimization.

**Disclosure of Interest:**

None Declared

